# Supplementary material for: Global landscape analysis of no-fault compensation programmes for vaccine injuries: A review and survey of implementing countries
Source: PLoS One. 2020 May 21;15(5):e0233334. doi: 10.1371/journal.pone.0233334 (PMC7241762; doi:10.1371/journal.pone.0233334)
Supplement: S1 File — (DOCX) [file pone.0233334.s001.docx]

Appendix 1: Search strategy for landscape analysis in PUBMED.

| Search term (PUBMED) | Items Found | Search input in PubMed |
| --- | --- | --- |
| Vaccine Injury: | 29004 | ("vaccines"[MeSH Terms] OR "vaccines"[All Fields] OR "vaccine"[All Fields]) AND ("wounds and injuries"[MeSH Terms] OR ("wounds"[All Fields] AND "injuries"[All Fields]) OR "wounds and injuries"[All Fields] OR "injury"[All Fields]) |
| Vaccine AND injury AND Compensation | 1751 | (("vaccines"[MeSH Terms] OR "vaccines"[All Fields] OR "vaccine"[All Fields]) AND ("wounds and injuries"[MeSH Terms] OR ("wounds"[All Fields] AND "injuries"[All Fields]) OR "wounds and injuries"[All Fields] OR "injury"[All Fields])) AND ("compensation and redress"[MeSH Terms] OR ("compensation"[All Fields] AND "redress"[All Fields]) OR "compensation and redress"[All Fields] OR "compensation"[All Fields]) |
| Vaccine injury AND compensation: | 1751 | (("vaccines"[MeSH Terms] OR "vaccines"[All Fields] OR "vaccine"[All Fields]) AND ("wounds and injuries"[MeSH Terms] OR ("wounds"[All Fields] AND "injuries"[All Fields]) OR "wounds and injuries"[All Fields] OR "injury"[All Fields])) AND ("compensation and redress"[MeSH Terms] OR ("compensation"[All Fields] AND "redress"[All Fields]) OR "compensation and redress"[All Fields] OR "compensation"[All Fields]) |
| ("vaccine injury") AND international | 82 | "vaccine injury"[All Fields] AND international [All Fields] |
| Vaccine injury AND (country name indicating they implement VICP) | 557 (USA) | ((("vaccines"[MeSH Terms] OR "vaccines"[All Fields] OR "vaccine"[All Fields]) AND ("wounds and injuries"[MeSH Terms] OR ("wounds"[All Fields] AND "injuries"[All Fields]) OR "wounds and injuries"[All Fields] OR "injury"[All Fields])) AND USA[All Fields]) AND ("compensation and redress"[MeSH Terms] OR ("compensation"[All Fields] AND "redress"[All Fields]) OR "compensation and redress"[All Fields] OR "compensation"[All Fields]) |
| ("vaccine damage") AND compensation | 43 | "vaccine damage"[All Fields] AND ("compensation and redress"[MeSH Terms] OR ("compensation"[All Fields] AND "redress"[All Fields]) OR "compensation and redress"[All Fields] OR "compensation"[All Fields]) |
| ((vaccine injury AND (“2010/01/01"[PDat] : "3000/12/31"[PDat] ))) AND compensation | 798 | ((("vaccines"[MeSH Terms] OR "vaccines"[All Fields] OR "vaccine"[All Fields]) AND ("wounds and injuries"[MeSH Terms] OR ("wounds"[All Fields] AND "injuries"[All Fields]) OR "wounds and injuries"[All Fields] OR "injury"[All Fields])) AND ("2010/01/01"[PubDate] : "3000/12/31"[PubDate])) AND ("compensation and redress"[MeSH Terms] OR ("compensation"[All Fields] AND "redress"[All Fields]) OR "compensation and redress"[All Fields] OR "compensation"[All Fields]) AND ("2010/01/01"[PubDate] : "3000/12/31"[PubDate]) |
| ((vaccine injury) AND Iatrogenic) AND liability | 18 (from 2010) | ((("vaccines"[MeSH Terms] OR "vaccines"[All Fields] OR "vaccine"[All Fields]) AND ("wounds and injuries"[MeSH Terms] OR ("wounds"[All Fields] AND "injuries"[All Fields]) OR "wounds and injuries"[All Fields] OR "injury"[All Fields])) AND Iatrogenic [All Fields]) AND liability[All Fields] AND ("2010/01/01"[PubDate] : "3000/12/31"[PubDate]) |
| Vaccine injury compensation | 49 from 2010 (41 relevant from skimming through publication titles) | (("vaccines"[MeSH Terms] OR "vaccines"[All Fields] OR "vaccine"[All Fields]) AND ("wounds and injuries"[MeSH Terms] OR ("wounds"[All Fields] AND "injuries"[All Fields]) OR "wounds and injuries"[All Fields] OR "injury"[All Fields]) AND ("compensation and redress"[MeSH Terms] OR ("compensation"[All Fields] AND "redress"[All Fields]) OR "compensation and redress"[All Fields] OR "compensation"[All Fields])) AND ("2009/12/31"[PDAT] : "3000/12/31"[PDAT]) |
| (no-fault compensation programs) AND vaccine injury AND (("2009/12/31"[PDat]: "3000/12/31"[PDat])) | 17 from 2010 (some already included in searches above, 11 articles look relevant from titles) | (no-fault[All Fields] AND ("compensation and redress"[MeSH Terms] OR ("compensation"[All Fields] AND "redress"[All Fields]) OR "compensation and redress"[All Fields] OR "compensation"[All Fields]) AND programs[All Fields]) AND (("vaccines"[MeSH Terms] OR "vaccines"[All Fields] OR "vaccine"[All Fields]) AND ("wounds and injuries"[MeSH Terms] OR ("wounds"[All Fields] AND "injuries"[All Fields]) OR "wounds and injuries"[All Fields] OR "injury"[All Fields])) AND ("2009/12/31"[PubDate] : "3000/12/31"[PubDate]) |
