# Supplementary material for: Global landscape analysis of no-fault compensation programmes for vaccine injuries: A review and survey of implementing countries
Source: PLoS One. 2020 May 21;15(5):e0233334. doi: 10.1371/journal.pone.0233334 (PMC7241762; doi:10.1371/journal.pone.0233334)
Supplement: S2 File — (DOCX) [file pone.0233334.s002.docx]

Supporting information: Profile of survey respondent.

| Country | Organization | Official Designation of respondent |
| --- | --- | --- |
| **America region** | | |
| **United states of America** | Division of Injury Compensation Programs/HRSA | Division Director |
| **Province of Quebec** | Ministry of Health and Social Services, Quebec | Deputy Director General of Public Health Protection |
| **European region** | | |
| **Austria** | Federal Ministry Labour, Social Affairs, Health and Consumer Protection | Head of Department |
| **Denmark** | (Danish) Patient Compensation Association | Chief of Staff |
| **Finland** | Finnish Pharmaceutical Insurance Pool | Director of Legislation |
| **France** | Ministry of Solidarity and Health - Directorate General of Health | Head of User Rights and Legal and Ethical Affairs Division |
| **Germany** | Robert Koch Institute, Berlin, Germany. National Public Health Institute | Head of Immunization Unit |
| **Hungary** | Department of Hospital Hygiene and Communicable Disease Control Ministry of Human Capacities | Head of Department |
| **Iceland** | No information | No information |
| **Italy** | Health Ministry – Public Administration | Public Manager |
| **Latvia** | Ministry of Health | Head of Legal Department |
| **Luxembourg** | Directorate of Health, public administration | Director of Health CMO |
| **Norway** | Norwegian Institute of Public Health | Senior Adviser; team leader for National Pharmacovigilance Center for Vaccines |
| **Russia*** | No information | No information |
| **Slovenia** | University Medical Centre (retired in 2013) | Head of Committee on Causality Assessment |
| **Sweden** | Public Health Agency of Sweden | Program manager |
| **Switzerland** | Federal Office of Public Health (BAG - OFSP - FOPH) | Head, Infection Control & Vaccination Programmes |
| **United Kingdom** | Department of Health and Social Care - Government Department | Immunization Policy and Strategy Manager (excluding flu) |
| **South East Asia region** | | |
| **Nepal*** | World Health Organization - Nepal | Team lead IPD Office Kathmandu |
| **Thailand*** | No Information | No Information |
| **Western Pacific region** | | |
| **China** | WHO China Office | National Professional Officer, EPI team |
| **Japan** | Ministry of Health, Labour and Welfare of Japan | Unit Chief Health Service Bureau Vaccination team |
| **New Zealand** | Accident Compensation Corporation Personal Injury Insurance | Chief Clinical Advisor |
| **Republic of Korea** | Korea Centers for Disease Control and Prevention (KCDC) | Epidemic intelligence service officer for AEFI |
| **Viet Nam*** | General Department of Preventive Medicine, Ministry of Health of Viet Nam | Vice Head of Division of Immunization and Biosafety Management |

*New compensation programs or programs not included in previous reviews
