# Supplementary material for: Global landscape analysis of no-fault compensation programmes for vaccine injuries: A review and survey of implementing countries
Source: PLoS One. 2020 May 21;15(5):e0233334. doi: 10.1371/journal.pone.0233334 (PMC7241762; doi:10.1371/journal.pone.0233334)
Supplement: S4 File — (PDF) [file pone.0233334.s004.pdf]

Informed Consent

Study title:

Evaluation of current policies and practices of no-fault compensation programs for vaccine related injuries in World Health Organization Member States.

Summary:

Vaccines are widely used in healthy populations to prevent diseases. Despite being extremely safe, vaccines are rarely associated with serious adverse reactions which may result in physical injuries, illnesses, disabilities and/or death. Considering the benefits of vaccination to society beyond protection of individuals from diseases, deliberate efforts by policy makers as custodians of public health are warranted, to address social concerns of vaccine injuries and to maintain confidence in vaccination programs. In some jurisdictions, no-fault compensation programs are established to waive the need for litigation, and to compensate individuals who suffer from a vaccine related injury due to the inherent risk of vaccination (e.g. neurological disorder in a child following vaccination with a well manufactured and administered vaccine). These programs do not require a claimant (individual with a vaccine injury or their legal representative) to prove negligence, or fault by the vaccine provider, health care system or the manufacturer prior to compensation.

Purpose:

This work is sponsored by the World Health Organization (WHO) Department of Essential Medicines and Health Products, and is intended to assess the current policies and practices of no-fault compensation programs for vaccine related injuries (referred to as Vaccine Injury Compensation Programs, VICP) in WHO member states. The data collected using this questionnaire will be property of WHO and will be used as reference material to guide evidence based policy formulation. This data will also be used for academic purposes as part of a thesis contributing to the award of a Master in Vaccinology for the investigator.

Study participation

You are being invited to complete a questionnaire that will take you approximately 30 minutes. You may be invited for a follow up interview via telephone or Skype for further clarifications. The survey questions aim to assess how vaccine injuries are compensated in your country. You may discuss the information being requested in the questionnaire and seek feedback or clarification from your team of other senior colleagues.

The same survey will be administered to all WHO member states that have a Vaccine Injury Compensation System in place.

All the responses provided will be confidential. However, the questionnaire will request for your contact details including email address and/or telephone number. To protect your privacy, all the information collected will be kept under password protection with limited access. The data with contact details will only be accessible to the student investigator, WHO and the study supervisor at the University of Siena. The data will be shared through publication in a peer reviewed journal and/or conferences, and will not include any details that may specifically identify you as the source of the information.

Participation in this survey is voluntary and you may decide to stop at any time. There will be no costs to you or payments made for your participation.

You are free to contact Dr Randy MUNGWIRA, mungwirar@who.int to clarify any question you might have regarding this survey,

I have read the foregoing information. I have had the opportunity to ask questions about it and all questions have been answered to my satisfaction. I consent voluntarily to be a participant in this survey.

Name of Participant: ____

Signature of Participant: ____

Date (dd/mmm/yyyy): ________________

QUESTIONNAIRE

Study title:

Evaluation of current policies and practices of no-fault compensation programs for vaccine related injuries in World Health Organization Member States.

Respondent's details:

Participant Identification number: ____

Country: ___________________________ Date (dd/mmm/yyyy): __________________

Organization (Name):____

Organization (Type):____

Designation (Official position): ____

Email: ______________________________ Telephone: ________________________

Section A: Administrative and Funding

1. When was Vaccine Injury Compensation Program (VICP) implemented in your country (year)? ______________________

2. At what level is your VICP administered/managed? (select all that applies)
   - ☐ Central or Federal government (national level)
   - ☐ State or Province government
   - ☐ City or municipal government
   - ☐ Private sector (e.g. pharmaceutical sector, insurance sector)
   - ☐ Other (Specify):____

3. How is the VICP in your country financed? (select all that applies)
   - ☐ Government or federal funding only
   - ☐ Levies (taxes) from pharmaceutical companies

- ☐ Covered by public insurance e.g. national health insurance
- ☐ Covered by private insurance
- ☐ As part of a general no-fault compensation program for all medicinal products
- ☐ Pharmaceutical industry direct funding
- ☐ As part of accidents compensation schemes
- ☐ Other (Specify any other source of funding e.g. other government partners or special insurance organizations not listed above): ____

Section B: Eligibility

4. What vaccines are eligible for compensation in your country's VICP? (select all that applies)
   - ☐ All mandatory vaccines
   - ☐ Specific mandatory vaccines (Please list covered mandatory vaccines): ____
   - ☐ All registered vaccines in your country (mandatory and non-mandatory vaccines)
   - ☐ Routine vaccines for children and pregnant women
   - ☐ Routine vaccines for children only
   - ☐ Recommended vaccines for adults e.g. influenza vaccines
   - ☐ Recommended vaccines based on indication e.g. travel
   - ☐ Recommended vaccines based on occupation e.g. health care workers and armed forces
   - ☐ Other (Please describe in details other categories of vaccines considered for compensation in your country): ____

5. Vaccine related injuries that are eligible for compensation in your country come from which healthcare sector?
   - ☐ Public sector

- ☐ Private sector
- ☐ Both public and private sectors

6. Who is eligible to receive compensation in your country?
   - ☐ Citizens only
   - ☐ State/province residents only
   - ☐ All including non-citizens
   - ☐ Other (specify): ____

7. Is there a time limit between vaccination and filing a vaccine related injury claim:
   - ☐ Yes
     - i. If Yes, please specify: ________________________________
   - ☐ No
8. What threshold is used to determine if a vaccine associated injury is eligible for compensation? (select all that applies)
   - ☐ Serious adverse events following vaccination
   - ☐ Disability as per predefined criteria
   - ☐ Any injury exceeding normal post vaccination reaction
   - ☐ None
   - ☐ Other (specify): ____
9. Does your VICP compensate vaccine injuries secondary to negligence (e.g. vaccine quality related reactions or immunization errors)
   - ☐ Yes
   - ☐ No

Comment:____

Section C: Process and decision making

10. How are vaccine related injuries reported or identified for compensation? (select all that applies)
    - ☐ Health care professional reporting to the VICP officials
    - ☐ Injured party filing a petition with the VICP

- ☐ Injured party filing complaint through a legal representative (lawyer)
- ☐ Other (Specify):____

11. Please describe the process and those involved indecision making from filing of a complaint to compensation of a petitioner: (Please provide a link or additional documents, if available, that may assist with further clarity of this process)

    ____

    ____

12. How long does it take to process a claim once it is filed? ________________

Section D: Standard of proof

13. Does your compensation scheme require evidence of a causal association between vaccine and an injury (standard of proof) before compensating claimants?
    - ☐ Yes
    - ☐ No (if no, proceed to question 16)

14. If yes, please specify which causality assessment outcome categories are compensated (select all that applies):
    - ☐ A. Consistent causal association to immunization
    - ☐ B. Indeterminate
    - ☐ C. Inconsistent causal association to immunization
    - ☐ D. Unclassifiable events
    - ☐ Other (Specify)
15. Who is involved in establishing causal association between a reported injury and vaccination prior to compensation? (select all that applies)
    - ☐ The reviewing physician
    - ☐ A special AEFI causality assessment committee
    - ☐ Special court of law
    - ☐ Other (Specify): ____

Section E: Elements of compensation

16. How are claimants compensated? (select all that applies)
    - ☐ Once off lump-sum of money
    - ☐ Monetary – calculated based on medical care costs and expenses, loss of earnings or earning capacity
    - ☐ Non- monetary terms – calculated based on pain and suffering, emotional distress, permanent impairment or loss of function
    - ☐ Other (Specify): ____

17. What category of benefits are offered as part of compensation in your country (Select all that applies)
    - ☐ Medical costs
    - ☐ Disability pension
    - ☐ Coverage of non-economic loss (pain and suffering, compensation to family)
    - ☐ Death benefits
    - ☐ Other (specify): ____

18. Are the financing categories selected above applied in a standard manner across all vaccine injury petitioners?
    - ☐ Yes, there are predefined standards for compensation
    - ☐ No, it depends on a case by case basis

Section F: Litigation rights

19. What options do claimants have for filing their vaccine related injuries in your country?
    - ☐ Vaccine injury compensation scheme alone
    - ☐ Both vaccine compensation schemes and tort law (civil claims are allowed)

i. If both, please clarify the circumstances when claimants are allowed to pursue both options :____________________________

____

Section G: Other information

20. How many petitions (claims for vaccine related injuries) have been filed in the last calendar year? ____________

21. Of the filed petition in question 19, how many were compensated?

____

22. How long does it take for a claimant to be compensated after filing a claim?

____

23. What would you consider as benefits of VICP for your country?

Item	Benefit	1	2	3	4	5
I.	Fair compensation for individuals inadvertently injured by vaccine meant for public good					
II.	Increase confidence in public vaccination program					
III.	Ensure adequate and sustained supply of vaccines					
IV.	Stabilize cost of vaccine					
V.	Protect manufacturers from liability					

VI.	Other (specify): ________________________________

Note: 1: Strongly disagree, 2: Disagree, 3: Neutral, 4: Agree 5: Strongly Agree

24. What are the challenges with the VICP in your country (select all that applies)?
    - ☐ Access to VICP services for individuals with vaccine related injuries
    - ☐ Long timelines for petitioners to receive compensation after filing claims
    - ☐ Inadequate funding for operations of the VICP
    - ☐ High volumes of claims overwhelming the financial capacity of the VICP
    - ☐ Narrow scope of eligibility of vaccine related injury qualifying for compensation
    - ☐ Strict requirements to establish causal relationship prior to compensation
    - ☐ Non-standardized compensation calculation
    - ☐ Complex and long bureaucratic administrative processes
    - ☐ Political interference with implementation of VICP
    - ☐ Lack of awareness of VICP existence by the public or communities
    - ☐ Others (Please describe any other challenges not listed above):

      ____

25. In your opinion, has the VICP of your country achieved its purpose since establishment?
    - ☐ Yes
    - ☐ No

    Please comment: ________________________________

26. Do you measure the effectiveness of the VICP in your country?
    - ☐ Yes
        i. If yes, please specify how your compensation program effectiveness is conducted:________________________________
    - ☐ No

27. What would you consider as appropriate criteria for assessing the effectiveness of VICP? (select all that applies)
    - ☐ Well-funded
    - ☐ Timely compensation of vaccine related injuries
    - ☐ Clear administrative procedures accessible to public
    - ☐ Broader scope of eligibility criteria for compensation
    - ☐ Equitable compensation across populations of a nation
    - ☐ Standard compensation calculation procedures
    - ☐ Community involvement in determining fair compensation rates before implementing VICP policy
    - ☐ Others (Specify): ____

28. In your opinion, would it be useful for WHO to develop guidance document to support countries in establishing vaccine injury compensation scheme?
    - ☐ Yes
    - ☐ No

29. Do you have similar compensation programs for injuries secondary to other medical products (i.e. drugs and medical devices)
    - ☐ Yes
    - ☐ No

30. Any other comments (Please provide a link or additional documents, if available, that may assist with further information on your compensation program):

    ____

    ____

Thank you for taking your time and responding to this questionnaire. We will share with you the study results, as soon as the study is completed.

Definitions:

Vaccine Injury Compensation Program: no-fault compensation systems implemented to compensate individuals who experience injuries following vaccination with well manufactured and administered vaccines.

No-fault compensation system: compensation schemes that do not require a claimant (victim or legal representative of a victim of a vaccine injury) to demonstrate malpractice or intention of malice by the health care provider or vaccine manufacturer in order to be compensated

Routine vaccines: vaccines that are recommended to individuals based on age and vaccination history e.g. vaccines included in the national immunization programs.

Recommended vaccines: Vaccines that are recommended by physician but are not part of routine or mandatory vaccination e.g. travel vaccines

Mandatory vaccines: vaccines which are obligatory or required by the law of a particular jurisdiction

Adverse Events Following Immunization (AEFI): is any untoward medical occurrence which follows immunization and which does not necessarily have a causal relationship with the usage of the vaccine

Serious AEFI: An AEFI is considered *serious*, if it:

- results in death,
- is life-threatening,
- requires in-patient hospitalization or prolongation of existing hospitalization,
- results in persistent or significant disability/incapacity,
- is a congenital anomaly/birth defect, or
- requires intervention to prevent permanent impairment or damage

Tort law: in common law jurisdictions, is a civil wrong that causes someone else to suffer loss or harm resulting in legal liability for the person who commits the tortious act.
