# Supplementary material for: Global landscape analysis of no-fault compensation programmes for vaccine injuries: A review and survey of implementing countries
Source: PLoS One. 2020 May 21;15(5):e0233334. doi: 10.1371/journal.pone.0233334 (PMC7241762; doi:10.1371/journal.pone.0233334)
Supplement: S5 File — (PDF) [file pone.0233334.s005.pdf]

Consentement éclairé

Titre de l'étude:

Évaluation des politiques et des pratiques actuelles des programmes d'indemnisation sans égard à la responsabilité pour les dommages liés aux vaccins dans les États membres de l'Organisation mondiale de la santé..

Résumé :

Les vaccins sont employés couramment chez des populations saines pour prévenir les maladies. Bien qu'ils soient extrêmement sûrs, les vaccins sont rarement associés à des réactions indésirables graves pouvant entraîner des blessures physiques, maladies, handicaps et/ou la mort. Considérant les avantages de la vaccination pour la société au-delà de la protection individus contre les maladies, les décideurs doivent veiller à à répondre aux préoccupations sociales liées aux vaccins et à maintenir la confiance dans les programmes de vaccination pour protéger la santé publique,.. Dans certains pays, des programmes d'indemnisation sans égard à la faute sont établis pour éviter les litiges et pour indemniser les personnes qui souffrent d'une blessure liée à un vaccin en raison du risque inhérent à la vaccination (par exemple. trouble neurologique chez un enfant après la vaccination avec un vaccin bien fabriqué et administré). Ces programmes n'exigent pas que le demandeur (personne avec une blessure après vaccination ou son représentant légal) prouve la négligence, ou la faute par le fournisseur du vaccin, le système de soins de santé ou le fabricant avant compensation.

But :

Cette enquête, initiée par le Département des médicaments essentiels et des produits de santé de l'Organisation mondiale de la Santé (OMS, vise à évaluer les politiques et pratiques actuelles des programmes d'indemnisation sans égard à la responsabilité pour les dommages liés aux vaccins (VICP) dans les Etats membres de l'OMS.. Les données recueillies à l'aide de ce questionnaire seront la propriété de l'OMS et seront utilisées comme matériel de référence pour guider la formulation de politiques fondées sur des preuves. Ces données seront également utilisées à des fins académiques dans le cadre d'une thèse contribuant à l'attribution d'un Master en vaccinologie pour l'investigateur.

Participation à l'étude

Vous êtes invités à remplir un questionnaire qui vous prendra environ 30 minutes. Vous pouvez être invité à un entretien de suivi par téléphone ou Skype pour plus de précisions. Les questions de l'enquête visent à évaluer comment les dommages liés aux vaccins sont compensés dans votre pays. Vous pouvez discuter de l'information demandée dans le questionnaire et demander des commentaires ou des éclaircissements à votre équipe ou d'autres collègues. La même enquête sera administrée à tous les États membres qui ont mis en place un programme d'indemnisation sans égard à la responsabilité pour les dommages liés aux vaccins.

Toutes les réponses fournies seront confidentielles. Cependant, le questionnaire vous demandera vos coordonnées, y compris votre numéro de téléphone et / ou votre adresse e-mail. Pour protéger la confidentialité, toutes les informations recueillies seront conservées sous la protection d'un mot de passe avec un accès limité. Les données avec vos coordonnées seront accessibles uniquement à l'investigateur, à l'OMS et au superviseur de l'étudiant investigateur de l'Université de Sienne. Les données seront partagées par le biais d'une publication dans une revue évaluée par des pairs et / ou des conférences, et n'incluront aucun détail qui pourrait vous identifier spécifiquement comme la source de l'information.

La participation à cette enquête est facultative et vous pouvez décider d'arrêter à tout moment. Il n'y aura pas de frais pour vous ou de paiements effectués pour votre participation.

Vous êtes libre de contacter le Dr Randy MUNGWIRA, mungwirar@who.int afin de clarifier toute question que vous pourriez avoir au sujet de cette enquête.

J'ai lu les informations ci-dessus. J'ai eu l'occasion de poser des questions à ce sujet et toutes les questions ont été répondues à ma satisfaction. Je consens volontairement à participer à cette enquête.

Nom du participant: ______________________________

Signature du participant: ______________________________

Date (dd/mmm/yyyy): ______________

QUESTIONNAIRE

Détails du répondant:

Pays du répondant: ______________________________

Nom de l'organisation: ______________________________

Type d'organisation: ______________________________

Désignation (poste officiel): ______________________________

Téléphone: ______________________________

Email: ______________________________

Section A: administratif et financement

1. Quand le Programme d'indemnisation des victimes de vaccination (VICP) a-t-il été mis en place dans votre pays (année)? ______________________________

2. A quel niveau votre VICP est-il administré et géré? (Cochez tout ce qui s'applique)

   - ☐ Gouvernement central ou fédéral (niveau national)
   - ☐ État ou Province or région
   - ☐ Ville ou municipalité
   - ☐ Secteur privé (p. ex., secteur pharmaceutique, secteur de l'assurance)
   - ☐ Autre (préciser) : ______________________________

3. Comment est financé le VICP dans votre pays ? (cochez tout ce qui s'applique)

 Gouvernement

 Prélèvements (impôts) des compagnies pharmaceutiques

 Couvert par l'assurance publique, par ex. assurance maladie nationale

 Couvert par une assurance privée

 Dans le cadre d'un programme générale d'indemnisation sans égard à la responsabilité pour tous les médicaments

 financement direct de l' industrie pharmaceutique

 Dans le cadre des régimes d'indemnisation des accidents

 Autre (précisez toute autre source de financement par exemple autres partenaires gouvernementaux ou des organismes d'assurance spéciales non répertorié ci-dessus):

____

Section b : admissibilité

4. Quels vaccins sont admissibles à une indemnité VICP dans votre pays ? (cochez tout ce qui s'applique)

 Tous les vaccins obligatoires

 Certains vaccins obligatoires (veuillez énumérer les vaccins obligatoires couverts): ____

 Tous les vaccins autorisée dans votre pays (vaccins obligatoires et non obligatoires)

 Les vaccins de routine pour les enfants et les femmes enceintes

 Les vaccins de routine pour enfants seulement

- ☐ Vaccins recommandés pour les adultes, par exemple les vaccins contre la grippe
- ☐ Vaccins recommandés selon indication par exemple voyage
- ☐ Vaccins recommandés selon le poste occupé sur l'occupation par exemple travailleurs de la santé et des forces armées
- ☐ Autre (préciser en détail les autres catégories de vaccins pris en considération pour l'indemnisation dans votre pays):

  ____

5. De quel secteur de la santé viennent les victimes de vaccination qui donnent lieu à indemnisation dans votre pays ?

   - ☐ Secteur public
   - ☐ Secteur privé
   - ☐ Des secteurs publics et privés

6. Qui est admissible à une indemnisation dans votre pays?

   - ☐ Citoyens uniquement
   - ☐ Résidents uniquement
   - ☐ Tous y compris les non-citoyens
   - ☐ Autre (préciser): ____

7. Y a-t-il un délai maximum entre la vaccination et le dépôt d'une demande d'indemnisation?

   - ☐ Oui

     i. Si oui, veuillez préciser le délai en mois ou en années: _______________

   - ☐ Non

8. Quel critère est utilisé pour déterminer si une blessure associée au vaccin est admissible à une indemnisation? (cochez tout ce qui s'applique)

☐ événement indésirable grave après vaccination

☐ Handicap selon les critères prédéfinis

☐ Toute blessure dépassant une reaction post-vaccinale normale

☐ Aucun seuil défini

☐ Autre (préciser): ____

9. Votre VICP indemnise-t-il les blessures par négligence liées au vaccin? (par exemple, réactions liées à la qualité du vaccin ou les erreurs de vaccination)

☐ Oui

☐ Non

Commentaire: ____

Section c: processus et prise de décision

10. Comment les dommages liés aux vaccins sont-elles signalées ou identifiées en vue d'une indemnisation? (Sélectionnez tout ce qui s'y rapporte)

☐ Un professionnel de santé informe les responsables du VICP

☐ Sujet Blessé dépose une requête auprès de la VICP

☐ Sujet blessé dépôse une plainte via un avocat

☐ Autre (préciser): ____

11. Veuillez décrire le processus et les personnes impliquées dans le processus de prise de décision, du dépôt d'une plainte à l'indemnisation du sujet blessé: (veuillez fournir un lien ou des documents supplémentaires, le cas échéant, qui pourraient aider à clarifier le processus)

____

____

____

____

12. Combien de temps faut-il pour traiter une demande une fois qu'elle a été déposée? ____

Section d : norme de preuve

13. Votre système d'indemnisation exige-t-il la preuve d'un lien de causalité entre la vaccination et la blessure (norme de preuve) avant d'indemniser les demandeurs?

 Oui

 Non (si non, passez à la question 16)

14. Dans l'affirmative, parmi les résulats d'évaluation de causalité ci-après, veuillez préciser lesquels sont indemnisés (Voir les définitions sur la dernière page; sélectionner tout ce qui s'applique):

 A. association causale cohérente avec la vaccination

 B. Indéterminé

 C. Association causale incohérente avec la vaccination

 D. Manifestations inclassables.

 Autre (préciser):____

15. Avant compensation, qui est impliqué dans l'établissement d'un lien de causalité entre une blessure signalée et la vaccination? (Cochez toutes les cases)

 Le médecin examinateur

 Un comité d'évaluation de la causalité des manifestations post-vaccinales indésirables

 Cour de justice spéciale

 Autre (préciser): ____

Section e : Eléments de compensation

16. Comment les demandeurs sont-ils indemnisés? (cochez tout ce qui s'applique)

 en une fois, avec une somme d'argent forfaitaire

 Monétaires – calculée en fonction des coûts des soins médicaux et frais, pertes de revenus ou de capacité de gain

 Conditions non monétaires - calculées en fonction de la douleur et de la souffrance, de la détresse émotionnelle, de la déficience permanente ou de la perte de fonction

 Autre (préciser): ____

17. Quels types de compensation sont accordés dans le cadre du programme d'indemnisation dans votre pays (sélectionnez toutes les réponses qui s'appliquent)

 Frais médicaux

 Pension d'invalidité

 Couverture des pertes non économiques (douleur et souffrance, compensation à la famille)

 Prestations de décès

 Autre (préciser): ____

18. Les types d'indemnisation sélectionnés ci-dessus s'appliquent-ils de façon standard à tous les demandeurs d'indemnisation pour les dommages liés aux vaccins?

 Oui, il y a des normes prédéfinies d'indemnisation

 Non, c'est évalué au cas par cas

Section F : droits contentieux

19. Quelles options les demandeurs ont-ils pour déposer leurs requêtes d'indemnisation pour dommages liés à des vaccins dans votre pays?

 Uniquement le système d'indemnisation des dommages liés au vaccin

 A la fois le système d'indemnisation des dommages liés au vaccin et la loi sur la responsabilité délictuelle (les demandes civiles sont autorisées)

i. Veuillez préciser les circonstances lorsque les demandeurs sont autorisés à poursuivre les deux options:

____

____

Section G: Autres informations

20. Combien de demandes d'indemnisation pour des dommages liés au vaccin ont été déposées lors de la dernière année civile?

____

21. Parmi les demandes déposées (indiquées question 20), combien ont été indemnisées?

____

22. Combien de temps cela prend-il pour être indemnisé après le dépôt d'une demande? ____

23. Quels sont selon vous les bénéfices du programme de VICP pour votre pays (sélectionnez une option par ligne)?

Article	Avantage	1	2	3	4	5
I.	Une juste compensation pour les personnes blessées par inadvertance par le vaccin, pour le bien public					
II.	Accroître la confiance dans le programme de vaccination publique					
III.	Assurer un approvisionnement suffisant et constant des vaccins					
IV.	Stabiliser le coût du vaccin					
V.	Protéger les fabricants de la responsabilité					
VI.	Autres (préciser): ________________________________					

Remarque: 1: fortement en désaccord, 2: pas d'accord, 3: neutre, 4: d'accord 5: entièrement d'accord

24. Quels sont les défis du VICP dans votre pays (sélectionner tout ce qui s'applique)?

- ☐ Accès aux services du VICP pour les personnes souffrant de dommages liés au vaccin
- ☐ Des délais longs pour que les demandeurs reçoivent une indemnisation après le dépôt des réclamations
- ☐ Financement inadéquate des opérations du VICP
- ☐ Des volumes élevés de demandes dépassant la capacité financière du VICP

□ Critères d'admissibilité restrictifs des dommages liés à un vaccin donnant droit à une indemnisation

□ Des exigences strictes pour établir le lien de causalité avant compensation

□ Calcul de l'indeminsation non standardisé

□ Des procédures administratives bureaucratiques, complexes et longues

□ Ingérence politique dans la mise en œuvre du VICP

□ Manque de connaissance du public ou des communautés de l'existence de VICP

□ Autre (veuillez décrire tout autre défi non mentionné ci-dessus):

____

25. Selon vous, le VICP de votre pays a-t-il atteint son objectif depuis sa création?

□ Oui

□ No

Commentez s'il vous plaît:

____

26. Mesurez-vous l'efficacité du VICP dans votre pays?

□ Oui

i. Si oui, veuillez préciser comment l'efficacité de votre programme d'indemnisation est mesurée:

____

□ Non

27. Que consideriez-vous comme des critères appropriés d'évaluation de l'efficacité des VICP ? (cochez tout ce qui s'applique)

- Bon financement
- Une compensation en temps opportun
- Procédures administratives claires et accessibles au public
- Critères d'admissibilité pour l'indemnisation plus large
- Une compensation équitable parmi les populations d'un pays
- Procédures standard pour le calcul de l'indemnisation
- Participation communautaire à la détermination de taux d'indemnisation équitables avant la mise en œuvre de la politique VICP
- Autres (préciser): ____

28. A votre avis, serait-il utile que l'OMS élabore un document d'orientation pour aider les pays à établir un régime d'indemnisation des dommages liés au vaccin?

- Oui
- Non

29 Avez-vous des programmes d'indemnisation similaires pour les dommages secondaires à d'autres produits médicaux (médicaments et dispositifs médicaux, par exemple)?

- Oui
- Non

30. Autres commentaires (veuillez fournir un lien ou des documents supplémentaires, le cas échéant, qui peuvent aider à comprendre votre programme d'indemnisation):

____

____

____

____

____

Merci d'avoir pris le temps de répondre à ce questionnaire. Nous partagerons avec vous les résultats de l'étude, dès que l'étude est terminée.

Définitions :

Programme d’indemnisation des dommages liés au vaccine (VCIP): système d’indemnisation sans faute mis en œuvre pour indemniser les personnes qui subissent des dommages suite à la vaccination avec avec un vaccin bien fabriqué et administré.

Régime d’indemnisation sans faute/san égard à la responsibilité : système d’indemnisation qui ne nécessite pas au demandeur (victime ou représentant légal d’une victime d’une blessure de vaccin) de démontrer la faute ou l’intention de la malveillance par le fournisseur de soins de santé ou un fabricant de vaccins afin d’être indemnisé

Vaccins de routine : vaccins recommandés aux personnes sleon leur âge et antécédent de vaccination (par exemple vaccins inclus dans les programmes nationaux de vaccination)

Vaccins recommandés : Les vaccins qui sont recommandés par le médecin, mais ne font pas partie des vaccins de routine (par exemple vaccination avant un voyage)

Vaccins obligatoires : les vaccins qui sont obligatoires en vertu de la loi d'une juridiction particulière

Manifestations postvaccinales indésirables – MAPI: est tout incident médical malencontreux qui fait suite à la vaccination et qui n’a pas nécessairement un lien de causalité avec l’utilisation du vaccin

Manifestations postvaccinales indésirables graves : une MAPI est considérée comme *grave*, si elle :

- entraîne la mort,
- met la vie en danger,
- nécessite une hospitalistaion ou prolongation d’hospitalisation,
- entraine une invalidité/incapacité persistante ou importante,
- consiste en une anomalie/malformation congénitale, ou
- nécessite une intervention pour éviter un des problèmes énumérés ci-dessus.

Responsabilité civile délictuelle : dans les juridictions de droit commun, il s'agit d'une faute civile qui fait que quelqu'un d'autre subit une perte ou un préjudice entraînant une responsabilité légale pour la personne qui commet l'acte délictueux.
