# Supplementary material for: Global landscape analysis of no-fault compensation programmes for vaccine injuries: A review and survey of implementing countries
Source: PLoS One. 2020 May 21;15(5):e0233334. doi: 10.1371/journal.pone.0233334 (PMC7241762; doi:10.1371/journal.pone.0233334)
Supplement: Attachment — Submitted filename: PLOS NFC.docx [file pone.0233334.s006.docx]

This article reports the results of a multi-pronged research project to find out which WHO member states have NFC and how those schemes operate. The article is well-written, well-researched, makes a large contribution to knowledge and considers the key implications of NFC schemes. It should be published subject to the following issues being addressed.

There appear to be two broad ways that governments can set up NFC schemes – either having purpose built schemes, or incorporating vaccine injuries as part of other compensation schemes, as in New Zealand. Looker and Kelly, in their study, only appeared to have looked for specific standalone schemes. We did the same in our study (Attwell, K., Drislane, S., and Leask, J. 2019. Mandatory vaccination and no fault vaccine injury compensation schemes: An identification of country-level policies. Vaccine 37 (1):2483-2348. doi: <https://doi.org/10.1016/j.vaccine.2019.03.065>.) which sought to identify only whether countries with mandatory vaccination also had NFC. (And judging by your results, it looks like we missed Latvia’s NFC scheme, although it looks like a ‘covers NFC by other means’ policy.)

This current study contributes way beyond this by having asked countries what they do (rather than just searching in the public domain) and it is this that appears to have allowed researchers who lack language and local policy knowledge to actually find out how local policies operate that may be missed by database and grey lit searches. This differentiation, and hence contribution, should be emphasised in the intro and the discussion, and maybe even the abstract. If this is the first time such detailed and triangulated methods have been used to explore who has NFC, then that should be stated.

That said, the limitation noted at line 339 now raises some confusion. It seems like maybe you specifically looked for NFC, but if you found something else that did the same job, you also included it. It would be worth noting in more detail in your methods and results what happened in this regard. For example, did your ‘screening’ of countries ask them in a binary fashion, or allow them to tell you if they had something that did the job (eg new Zealand). Or did you find out about cases like NZ from other literature? I think it is important for readers to better understand your methodology, and hence the limtations, so you should elaborate these processes.

Line 97 ‘all WHO member states were screened’ – what does this mean? Are you introducing the steps you describe in the following sentences, or was this ‘screening’ something else? It sounds like an in introduction, so maybe make this clear eg. ‘were screened using several methods…’ It sounds later (results) like you actually contacted every member state as part of this screening. If this is true, then say so back here at line 97. If not, then the first part of the Results needs rewording.

Line 107 maybe insert ‘with a no-fault scheme identified through these machanisms…’ and then continue the sentence with what the survey was seeking to find out.

Line 121 onwards – was the 151 feedback simply countries telling you that they did or didn’t have NFC, or other things? Please specify this more clearly. Were surveys and other data sources used in conjunction in all of the 23 programs you evaluated, or just in some? What determined whether you did or didn’t use government docs?

Line 172 – since Japan doesn’t have mandatory vax, consider rewording the entire sentence or leave Japan out of it (and put in own category).

Line 298. This appears to be the first time you told us you asked the participants this question. It’s so good that you did. Please tell us earlier in the article how you did and why you did. Likewise for their assessments of challenges. To this point, I was expecting that you’d just followed Evans’ structure. Better and more expansive questions also enhances the contribution of this paper and should be emphasised elsewhere in the text.

Line 302-enhance legal basis for mandatory vax systems – presumably only if they had such systems in place.
